# Death of the Inventor of the Hypodermic Syringe

**Published:** 1892-09

**Authors:** 


### Death of the Inventor of the Hypodermic Syringe.

The ordinary hypodermic syringe is known in France as the "*Seringe de Pravaz,*" the instrument having been invented by Dr. Pravaz, of Lyons. The death of this gentleman is announced in this week's journals. He was the director of an orthopedic establishment in the silk capital, and was well known as a *medicin orthopediste.*

